# Children in Workhouses

**Published:** 1913-11-22

**Authors:** Albert Spicer

**Affiliations:** Chairman; Vice-Chairman


### Children in Workhouses.

We have been requested to publish the following letter which has been sent by the State Children's Association to the President of the Local Government Board :—

Sir,—At the last meeting of the State Children's Association Executive Committee the Draft Poor-Law Institutions Order, in so far as it affects children, was considered. While rejoicing that the Order would make it impossible for Boards of Guardians to retain children permanently in workhouses, the committee deeply regret the proposal to fix the age until which children may be maintained in workhouses at five years. They desire to enter their earnest protest against this proposal on the following grounds :

1. Educationists assert that the years between three and five are of the utmost importance in the children's mental and moral development, for during that period, under proper training, formative habits of mind and body are acquired which largely determine the children's worth in after years.

2. During these highly impressionable years, therefore, it is essential that the children should have every reasonable opportunity of learning through educative play, through the handling of toys and articles suitable to their use, as well as by unconscious imitation of those surrounding them. In many workhouses these infants have insufficient toys or none at all. The officials charged with their oversight are unable to do more than attend to their physical needs and not infrequently are assisted, even in this particular, by pauper women. The monotony and inactivity of their meaningless existence and the lack of open-air life are pernicious to these unfortunate children.

3. While in the past Guardians have been in the habit of boarding out their children at an early age and of placing those of three years old in scattered homes—where their presence is both welcome and useful to the elder children—this proposal to make five years of age the limit up to which they may be retained amid workhouse surroundings will have the effect of making that age the minimum at which Guardians will plan other methods of upbringing. Since the appearance of the Draft Order a Board of Guardians in the West has been recommended by its Children's Committee not to remove the children from the workhouse to the scattered homes until five years old, instead of at three as heretofore. We fear that this example may be widely followed and that Boards of Guardians may think themselves justified in inferring that the Local Government Board has instigated this retrograde step.

4. At the age of three children frequently have to be removed from the workhouse nurseries, where their presence is detrimental to the infants of tenderer years. A change of surroundings being then necessary, it is a suitable time for the young life to be placed where it can, by attempt and experience learn how to live well.

For these weighty reasons we ask, therefore, that the limit of children's maintenance in workhouses be fixed at three years old and not at five as suggested, the children's welfare being in our view—and we are sure also in the view of your Department—above any question of "administrative convenience." We desire also to ask that the period allowed to Guardians in which to make new arrangements for their children's upbringing should be fixed at twelve months instead of two years as suggested. In the debates on the Local Government Board Vote the House of Commons, irrespective of party, has made it perfectly clear that it does not approve of retaining children in workhouses. Repeated warnings on this subject have for years been addressed by your Department to Boards of Guardians, no one of whom can plead ignorance of, or surprise at, the coming change. Article 4 of this Order will mainly affect the most backward Boards of Guardians, and we earnestly ask that the children in their care may not be consigned to two more years of workhouse life.—We are, Sir, your obedient servants,

(Signed) Lytton, Chairman.

Albert Spicer, Vice-Chairman.

